# COVID-19 and its impact on child and adolescent psychiatry – a German and personal perspective

**DOI:** 10.1017/ipm.2020.43

**Published:** 2020-05-14

**Authors:** Jörg M. Fegert, Ulrike M. E. Schulze

**Affiliations:** 1 Department of Child and Adolescent Psychiatry/Psychotherapy, University Hospital of Ulm, Ulm, Germany; 2 Center for Psychiatry, Department of Child and Adolescent Psychiatry and –Psychotherapy, Böblingen, Germany

**Keywords:** child protection, COVID-19, Germany, perspective, youth psychiatric care

## Abstract

As in other European countries, the current COVID-19 pandemic has not only massively restricted normal life in Germany but it is also having a significant effect on medical treatment, particularly in the areas of child and adolescent psychiatric care, as well as on university teaching. The federal structure of Germany and epidemiological differences between individual federal states have had a crucial impact on the regulations issued and their success. During the last number of weeks, tele-child-psychiatry and psychotherapy have increased, and outpatient services have been used cautiously and sparingly. Medical staff numbers will be augmented by doctors and nurses returning from retirement and also by medical students on a voluntary basis. The federal government has warned that discrepancies in education will increase due to the closure of schools. Questions of child protection are currently of particular importance in the context of such closures and the non-availability of day-care centers.

At the end of January 2020, the first COVID-19 cases were recorded in Europe; the first case in Germany was reported on 27th January. As in other countries, the initial reaction of the general public was denial: ‘It is a Chinese problem’, ‘It is an Italian problem’, ‘The virus is not dangerous for young people’. ‘Corona parties’ of teenagers and teens was one of the consequences of the initial phase of the crisis. But very soon, people understood that this pandemic might hit their own family – grandparents could die or a child with an underlying medical condition could die, despite its young age. As a result, personal contacts were reduced to a minimum. From a developmental perspective, adolescents and young adults seem to suffer increasingly from restricted social contacts. An advice regroup to the German Federal Ministry for Family Affairs, Senior Citizens, Women and Youth pointed out the special needs of adolescents in respect of peer group interaction from the developmental and educational perspective. Newspapers in German speaking countries reported on the special consequences of socialization for adolescents, for example, NZZ (https://www.nzz.ch/meinung/corona-krise-ein-albtraum-gerade-auch-fuer-jugendlicheld.1548710).

Under the German federal system, the chancellor and the federal government give general directions but policies are set at state level. This is an important consideration when assessing the effect of COVID-19 in different German states. For example, there is a huge epidemiological difference between states with lower numbers of infections and fatalities in the North, particularly in the Northeast. Ulm is located on the border between the German states of Baden-Württemberg and Bavaria. Both states have the highest caseload and have implemented very strict rules, while Mecklenburg-Western Pomerania, by far the least affected state, sealed itself off from the outside world by closing state borders.

On 16th March, the federal government announced a general lockdown. This has increased stress in families: parents were now forced to work from home as well as trying to simultaneously perform home schooling. The federal government warned that during the crisis educational disparities would increase (The Academic Advisory Board on Family Affairs, Germany 2020, https://www.hertie-school.org/en/debate/allcontent/detail/content/rethinking-germanys-2020-summer-vacation/). During the first weeks of the lockdown, there has also been a series of reports of increased domestic violence and child abuse (Fegert *et al*. 2020a).

As a consequence of the lockdown, the entire medical system has been reorganized with big differences noted on a nationwide level but also within federal states. The number of intensive care beds has rapidly increased, while some forms of high-risk treatments such as day-treatment units in child and adolescent psychiatry have been closed at the same time as school closures. In general, elective procedures are postponed whenever possible, but some patient groups (e.g. children with autism, children with intellectual disabilities, or comorbid conditions and cancer patients) have expressed concern about their continued care. Tele-child-psychiatry and psychotherapy have increased enormously, and the health care system has recognized and adapted to these new forms of service provision in a short space of time. Retired doctors and nurses have come back into the health service, and many medical students and student nurses have volunteered to work in ‘drive-in’-corona-testing stations. Students also volunteer on the hospital wards or in specific medical facilities (e.g. dialysis facilities) to ensure basic care is maintained for all patients. Volunteers have also been recruited by the medical chambers and professional associations to man hotlines and to provide psychosocial support for medical staff.

What is the situation pertaining to child and adolescent psychiatry? What we appear to be witnessing during March and April is the ‘calm before the storm’. But there are huge differences on a European level and in Germany between different states and within states (see ESCAP policy statement Fegert *et al*. 2020b). For example, in some university hospitals, wards have been closed in the context of the reorganization of hospitals to provide more intensive care beds, while, as is the case in some specialized psychiatric hospitals, the new hygiene measures could be implemented with little disruption of normal service provision, as they did not have to contribute as much to the reorganization. As different forms of outpatient treatment are transformed to telephone or video contacts, inpatient treatment is maintained in most locations. In some centers/clinics, quarantine conditions for new cases (e.g. following a suicide attempt) have been put in place. Visits to the wards have been drastically limited, physical distancing must be observed, and facemasks are mandatory on the wards. Nevertheless, the patience and willingness of patients and their families to cooperate with these strict measures has been admirable. Outpatient services are used cautiously, given the increased risk of infection. Most institutions have established a preclearance and screening operation by telephone to try to avoid any unnecessary physical contact in waiting rooms. A small number of patients and families have reacted negatively and aggressively to these new measures resulting in many hospitals employing extra security personnel.

The issue of child protection during the lockdown has become of particular concern. Due to the closure of schools, families at home are now ‘at each other’s mercy’. This may already be demanding at times when normal structures and peer relationships are intact, but it poses a considerable risk under the strained circumstances of the current crisis. Educational day care facilities are also closed, although physical contact is still maintained to a limited extent. Youth welfare outpatient services are largely suspended, again with greater use of telephone contact or video conferences.

Medical colleagues in hospitals and clinics have adapted to the unprecedented changes in an admirable way; daily adjustments are being made in order to deal effectively with a clinical situation that is constantly changing. Political authorities are being forced to make such changes on an almost weekly basis, and health care has been forced to react accordingly in rearranging service provision. The general managers of the hospitals and special Corona taskforces have been empowered to redeploy professionals working in our units to other areas of the hospital. While everything is focused on the COVID-19 cases, the budget for the maintenance of mental health services is not currently clear. Under normal circumstances, these are financed case by case, but this makes little sense during and after the present crisis. In order to maintain service quality and to support a return to normality, it is the opinion of the authors that the budget for the last 2 years before the crisis should be the basis for the financing of child and adolescent mental health care services in Germany to give them an opportunity to return to normal standards of service provision.

In Germany, the legal principle that mental illness should have parity with physical illness has been established since the 1970s. This demand was expressed in writing when, in September 1975, an expert commission appointed in August 1971, handed over the report of the ‘Psychiatrie-Enquête’ to the German Parliament. It took until 1988 for the law to reflect this expert opinion when a formal paragraph that pointed out the specific needs of patients with mental illness was finally introduced (§ 27 SGB V. The debate is documented in the protocols of the committee of social affairs of the German Bundestag).

The urgent needs of high-risk sections of the population during the current pandemic are clear. But the needs of child and adolescent psychiatrists in training should not be forgotten or neglected. Medical exams, the defence of doctoral theses, internal grand rounds, and student supervision have all been postponed or have been changed to teleconferences. There have been increased requests for e-learning programmes from child psychiatrists and psychologists/psychotherapists and social workers in quarantine. Within the first 2 weeks, more than 6000 health and social work professionals subscribed to these programmes. Our department has opened at its own cost all e-learning courses in child protection, trauma treatment, and transition psychiatry, and within the first few days, more than 2000 health professionals subscribed to these programs to earn CME points. The German Society of Child and Adolescent Psychiatry, Psychosomatics, and Psychotherapy supports this initiative. After the crisis, child and adolescent psychiatrists that have been transferred to other services should not encounter disadvantages with respect to their training. Moreover, the existing regulations governing child and adolescent psychiatry training will have to be adapted to prevent a lasting negative impact on the careers of future child and adolescent psychiatrists.
